# Bioupgrading of the aqueous phase of pyrolysis oil from lignocellulosic biomass: a platform for renewable chemicals and fuels from the whole fraction of biomass

**DOI:** 10.1186/s40643-023-00654-3

**Published:** 2023-05-26

**Authors:** Selim Ashoor, Tae Uk Khang, Young Hoon Lee, Ji Sung Hyung, Seo Young Choi, Sang Eun Lim, Jinwon Lee, Si Jae Park, Jeong-Geol Na

**Affiliations:** 1 Department of Agricultural Microbiology, Faculty of Agriculture, Ain Shams University, Hadayek Shoubra, Cairo, 11241 Egypt; 2 Department of Chemical and Biomolecular Engineering, Sogang University, Seoul, 04107 Republic of Korea; 3 Department of Chemical Engineering and Materials Science, Ewha Womans University, Seoul, 03760 Republic of Korea

**Keywords:** Lignocellulosic biomass, Pyrolysis oil, Aqueous phase, Biological conversion, Toxicity mitigation

## Abstract

Pyrolysis, a thermal decomposition without oxygen, is a promising technology for transportable liquids from whole fractions of lignocellulosic biomass. However, due to the hydrophilic products of pyrolysis, the liquid oils have undesirable physicochemical characteristics, thus requiring an additional upgrading process. Biological upgrading methods could address the drawbacks of pyrolysis by utilizing various hydrophilic compounds as carbon sources under mild conditions with low carbon footprints. Versatile chemicals, such as lipids, ethanol, and organic acids, could be produced through microbial assimilation of anhydrous sugars, organic acids, aldehydes, and phenolics in the hydrophilic fractions. The presence of various toxic compounds and the complex composition of the aqueous phase are the main challenges. In this review, the potential of bioconversion routes for upgrading the aqueous phase of pyrolysis oil is investigated with critical challenges and perspectives.

**Graphical Abstract:**

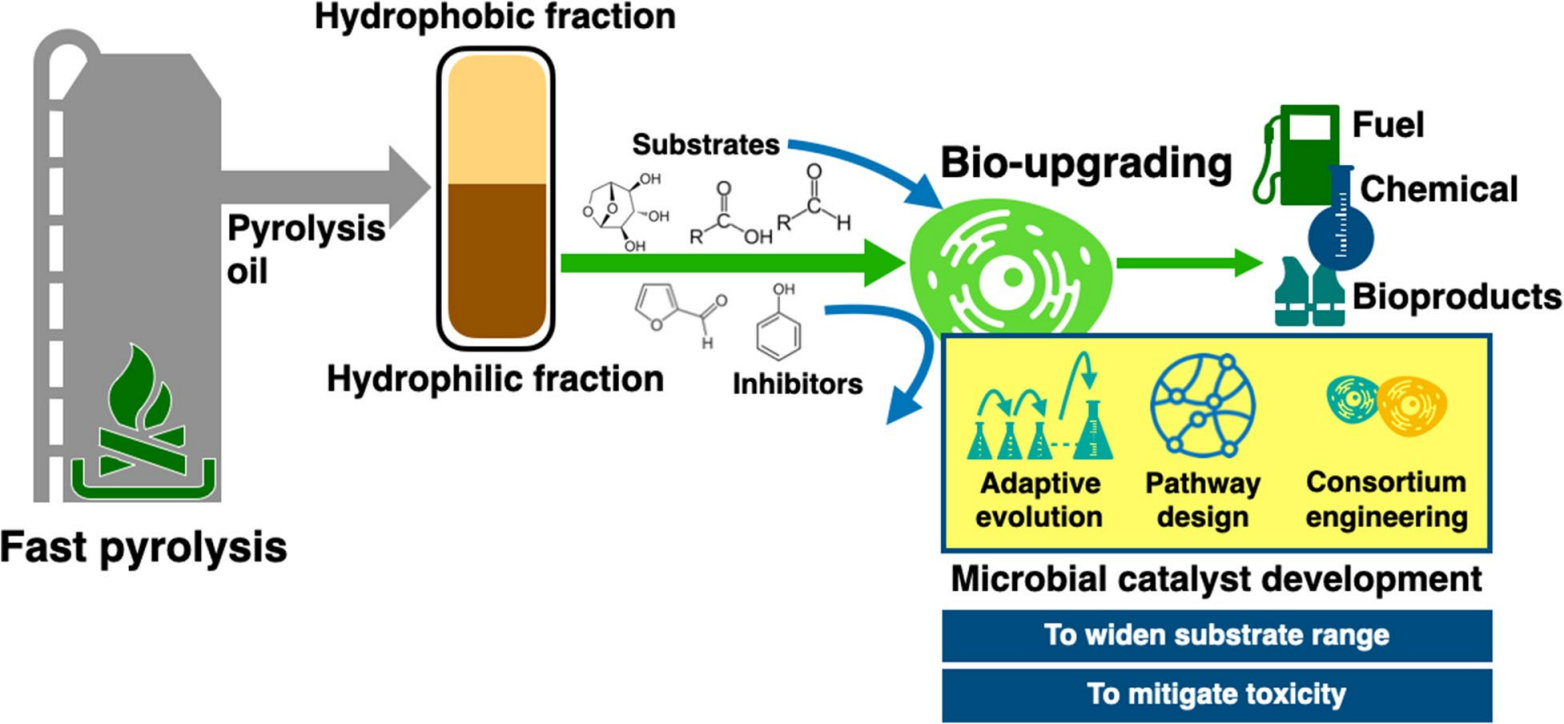

## Introduction

As the global concern caused by climate change becomes a reality, efforts to build a carbon-neutral society are accelerating. Countries worldwide have announced long-term LEDS (Low Emission Development Strategies) roadmaps (Waisman et al. 2019), and technical support for a sustainable and green society is required. Under this circumstance, it is crucial to secure energy and chemical production resources. Some technologies have already reached grid parity levels under a specific environment in the energy sector through continuous R&D investments. However, for producing liquid transportation fuels or chemicals containing carbon, biomass is the superior alternative in the short term until carbon dioxide (CO_2_) utilization technology is evaluated on a large scale (Moradian et al. 2021).

Biomass can be a carbon-neutral resource and is easy to obtain in bulk amounts from wood, grass, or agricultural wastes (Xu et al. 2020). Among the different biomass types, lignocellulosic biomass is a promising feedstock and one of the most plentiful materials in the world (Arnold et al. 2017). The first consideration in producing fuels and chemicals using lignocellulosic biomass is to maximize product yield without generating waste. The presence of non-fermentable fractions, including lignin, has a disadvantage in that the yield of the product is low when relying on biological conversion only (Nguyen et al. 2017; Lee et al. 2020). To utilize all components of biomass, thermochemical routes used in coal or petrochemical industries for ages have been tried (Kumari and Karmee 2022).

Pyrolysis converts biomass into liquid bio-oil, solid biochar, and non-condensable gas (Neumann et al. 2016; Palazzolo and Garcia-Perez 2021). Product traits from pyrolysis depend on the three principal components of biomass. Cellulose and hemicellulose are responsible for more bio-oil and syngas, while lignin makes more char content (Baloch et al. 2018). Fast pyrolysis has received much interest, since the process can produce considerable amounts of liquid bio-oils, which are transportable and versatile (Bridgwater 2017; Meier 2017). These bio-oils can be separated into the aqueous and organic phases. The organic phase can be directly upgraded to transport fuels (Mortensen et al. 2011; Bridgwater 2012; Wang et al. 2013), whereas the aqueous fraction requires additional upgrading for drop-in products adequate to the existing petroleum-based infrastructure. For this purpose, bioconversion technology can be an option; however, pyrolysis oils are not conventional substrates available to microorganisms. The main challenges are various toxic compounds and the complex composition of the aqueous phase (Islam et al. 2015; Arnold et al. 2017). Therefore, wild or genetically engineered microorganisms may be required to assimilate pyrolytic substrates and tolerate these toxic compounds. There have been comprehensive reports on strain improvement or toxicity mitigation strategies for pyrolysis oil as a promising feedstock for microbial transformation (Neumann et al. 2016; Arnold et al. 2017).

**Fig. 1:**
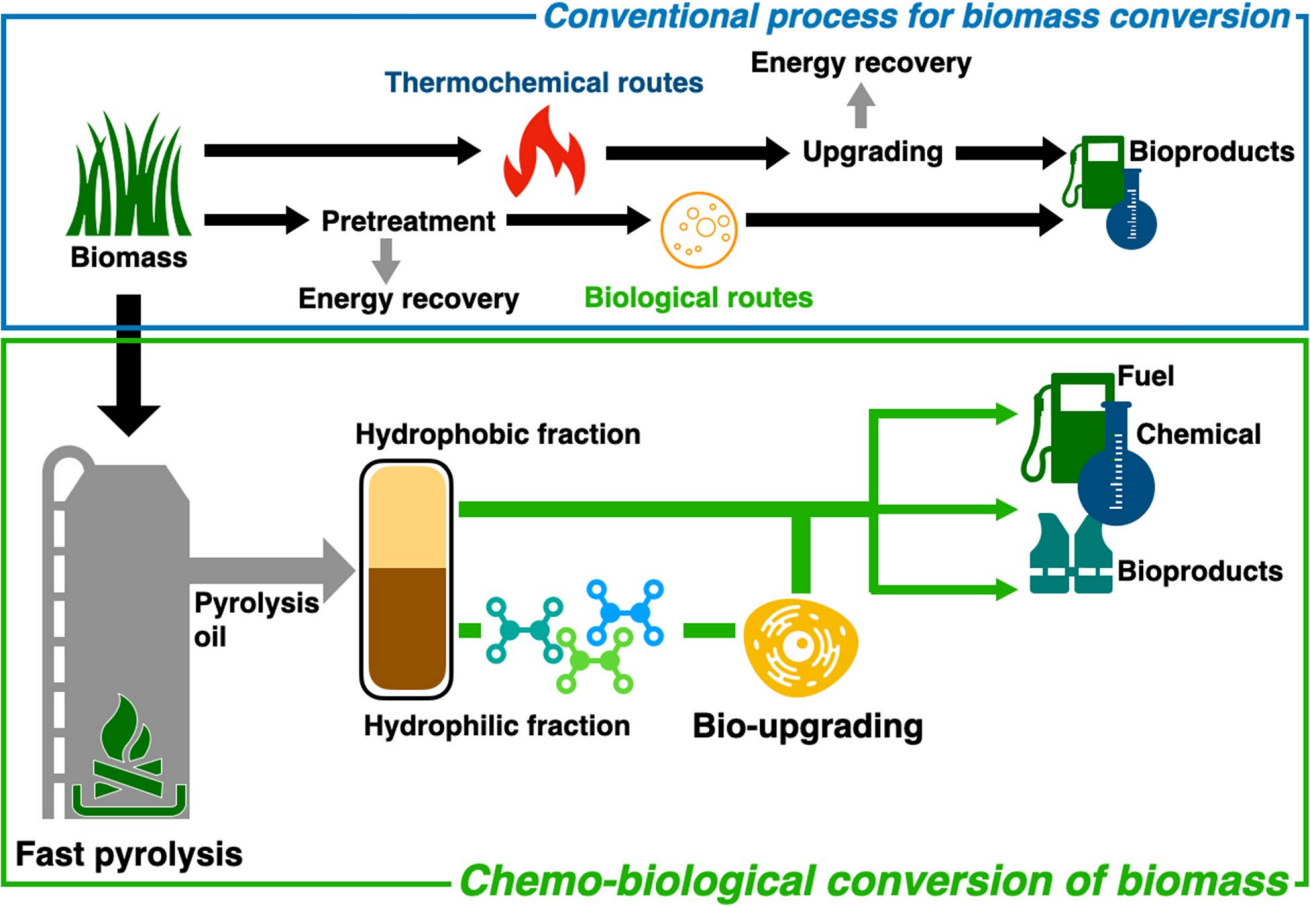
Process scheme for transforming lignocellulosic biomass into valuable products based on fast pyrolysis combined with biological upgrading

This review aims to introduce a hybrid approach that combines pyrolysis, a thermochemical conversion method, and biological upgrading to establish a carbon-neutral society based on biomass. This integration addresses the limitations of both technologies (Fig. 1). First, the review briefly summarizes the thermochemical conversion routes of biomass and presents the characteristics of pyrolysis technology. Subsequently, the physicochemical properties of the aqueous product of pyrolysis and the advantages of bioconversion technology for this fraction are discussed. Recent studies on the opportune production of various bioproducts from aqueous pyrolysates are explored, and strategies are proposed to overcome the challenges in bioupgrading technology. Therefore, this review presents the potential routes for microbial upgrading of the aqueous phase of pyrolysis oil into value-added products with an investigation of the current status, mitigating strategies for challenges, and future perspectives.

## Thermochemical biomass conversion processes

Various thermochemical processes can convert biomass into products like heat and electricity, biochar, gases, liquid fuels, and chemicals (Yerrayya et al. 2020). These technologies have different process conditions, such as temperature, pressure, and oxygen participation in the reaction, and could be appropriately applied depending on raw materials and target products.

Combustion is a simple way to obtain heat and electricity from the chemical energy present in biomass. This process involves burning lignocellulosic biomass at a high temperature (1000–1200 °C) in the presence of oxygen, forming carbon monoxide, carbon dioxide, water, ash, and heat energy (Bridgwater and Peacocke 2000; McKendry 2002; Demirbas 2004; Luthfi et al. 2022). Gasification is a process that converts biomass to syngas, which includes carbon monoxide, hydrogen, carbon dioxide, methane, nitrogen, and hydrocarbons. It can be performed at a high temperature up to 1400 ℃ with low/absent oxygen (Lee et al. 2019). Gasification is the most efficient technique for hydrogen production (Ahmad et al. 2016). Hydrothermal liquefaction transforms biomass into liquid products using subcritical water at intermediate temperatures (250–374 °C) with high pressures (40–220 bar). This technique can deal with wet biomass having high moisture content, thus reducing the cost of biomass drying (Lee et al. 2019).

On the other hand, pyrolysis is a thermochemical decomposition of biomass that can be performed at temperatures above 400 ℃ without oxygen to produce bio-oil, biochar, and non-condensable gases. These products are of great importance due to their potential use in different fields, making pyrolysis almost a zero-waste technique (Badger 2002; Bennett 2006; Tu et al. 2022).

Therefore, the unique strength of pyrolysis technology compared to other thermochemical methods is that it can produce versatile products under relatively mild conditions. In particular, it has a high yield of liquid products with excellent transport properties. Pyrolysis can be categorized into three types depending on operation conditions regarding reaction time and temperature (Yerrayya et al. 2020). These types are known as slow, intermediate, and fast pyrolysis (Baloch et al. 2018). Slow pyrolysis is performed at a temperature below 500 ℃ with a reaction time ranging from 10 to 2000 min, while intermediate pyrolysis has a temperature range of 400–500 ℃ and a reaction time of 1–10 min. Fast pyrolysis requires less reaction time (0.5—5 min) with a high reaction temperature (450–650 ℃) (Xu et al. 2020), which can prevent further breakdown of the products of the pyrolysis process into non-condensable compounds, thus increasing the contents of bio-oil in the final product (Dhyani and Bhaskar 2018). Table 1 compares different pyrolysis methods in terms of reaction conditions and primary target products, and we will mainly focus on fast pyrolysis and its product.

**Table 1:** Comparison of different types of pyrolysis

Type of pyrolysis	Slow	Intermediate	Fast	References
Reaction time (min)	10–2000	1–10	0.5–5	(Bridgwater 2012; Xu et al. 2020)
Heating rate (℃/s)	< 1	1–1000	~ 1000	
Reaction temperature (℃)	< 500	400–500	450–650	
Product yields (bio-oil, biochar, gas)	30 wt%, 35 wt%, 35 wt%	50 wt%, 25 wt%, 25 wt%	75 wt%, 12 wt%, 13 wt%	

## Bio-oil from fast pyrolysis and its upgrading

Fast pyrolysis can produce three types of products in different proportions: a liquid product known as bio-oil or pyrolysis oil, a solid product known as biochar, and a gaseous product known as syngas (Di Blasi et al. 1999; Mohan et al. 2006; Arnold et al. 2019b). Among the three products, the liquid product has advantages such as more accessible transport and storage and higher energy density than the other two products (Jena and Das 2011; Ahamed et al. 2021).

### Composition

Pyrolysis oil composition should be analyzed to facilitate its upgrading process. For that, several analytical methods are used, including gas chromatography (GC), two-dimensional GC (GC × GC), pyrolysis GC–mass spectrometry (Py-GC/MS), liquid chromatography (LC), high-resolution MS (HRMS), nuclear magnetic resonance (NMR), and Fourier transform infrared spectroscopy (FTIR). Through these methods, more than 400 compounds with different molecular sizes and functional groups are characterized (Mohan et al. 2006; Ioannidou et al. 2009; Meier et al. 2013; Staš et al. 2014), including anhydrosugars, acids, aldehydes, furans, phenols, lignin-derived compounds, and others (Brown 2007; Hu et al. 2011). Among the different compounds, levoglucosan is the most promising one. It is mainly generated from the pyrolysis of cellulose or starch. Since cellulose does not compete with the food supply, it is preferred over starch (Junior et al. 2020). Cellulose-rich fractions of different biomasses were used to obtain levoglucosan through fast pyrolysis. The maximum levoglucosan yield (59.8 wt%) was achieved using the cellulose-rich fraction of bagasse (Zheng et al. 2017). Significant compounds of pyrolysis oil and their compositions are summarized in Table 2.

**Table 2:** Major compounds of pyrolysis oil and their compositions

Type	Compound name	Minimum (wt%)	Maximum (wt%)	References
Sugars	Levoglucosan	0.1	59.8	(Milne et al. 1997; Demirbas 2009; Bertero et al. 2012; Staš et al. 2014; Zheng et al. 2017)
	Cellobiosan	0.4	3.3	
	Fructose	0.7	2.9	
Carboxylic acids	Acetic acid	0.5	17.0	
	Formic acid	0.3	9.1	
	Propionic acid	0.1	2.0	
Aldehydes	Glycolaldehyde	0.9	17.5	
	Acetaldehyde	0.1	8.5	
	Formaldehyde	0.1	3.3	
Alcohols	Methanol	0.4	8.2	
	Ethanol	0.5	3.5	
Ketones	Acetol	0.2	7.4	
Aryl-aldehydes (Furans)	Furfural	1.5	3.0	
Phenolic compounds	Phenol	0.1	3.8	
	Guaiacol	2.8	2.8	

### Physicochemical properties

The physicochemical properties of pyrolysis oil differ significantly from heavy fuel oil, a target product to be replaced. Pyrolysis oil has a much higher water and oxygen content than heavy fuel oil, leading to a low heating value (Czernik and Bridgwater 2004; Valle et al. 2019; Xu et al. 2020). The low pH of pyrolysis oil causes corrosion problems in handling equipment and storage vessels (Zhang et al. 2007; Dhyani and Bhaskar 2018). Therefore, pyrolysis oil upgrading is needed to improve its properties. Some physicochemical properties of pyrolysis oil are presented in Table 3.

**Table 3:** Typical physiochemical properties of pyrolysis oil

Property	Pyrolysis oil	Heavy fuel oil	References
Water (wt%)	15–30	0.1	(Oasmaa and Czernik 1999; Zhang et al. 2007; Valle et al. 2019)
pH	2–4	–	
Viscosity (centipoise, 50 °C)	40–100	180	
C (wt%)	48–65	83–86	
O (wt%)	30–45	< 1	
H (wt%)	5.5–7	11–14	
N (wt%)	0–0.2	0.3	
S (wt%)	< 0.05	< 3	

### Pyrolysis oil upgrading

As mentioned above, there are obstacles to using pyrolysis oil directly. Several methods have been developed for pyrolysis oil upgrading to improve its properties (Xu et al. 2020) by decreasing the boiling point or viscosity and removing impurities. Some of these methods are described as follows:

Emulsification is a simple method to blend pyrolysis oil with other fuel oil such as biodiesel or diesel in the presence of surfactants to enhance its properties for ignition and reduce its viscosity. The resulting mixture can be used as engine fuels with low emissions (Zhang et al. 2013; Liang et al. 2018). However, the high cost of surfactants and energy consumption limit large-scale implementations (Kanhounnon et al. 2019). In addition, since emulsification mainly focuses on improving combustion characteristics, it cannot contribute to producing renewable chemicals.

Catalytic cracking and hydrotreating improve the physical properties of pyrolysis oil by removing heteroatoms such as oxygen or nitrogen present in the oil molecules. Catalytic cracking can lower the boiling point and viscosity of the product through various pathways, including decomposition, decarboxylation, decarbonylation, and carbon rearrangement (Lian et al. 2017). However, this method may cause a decrease in the liquid product yield and require frequent catalyst replacement due to poisoning substances.

Hydrodeoxygenation is a way to remove oxygen from pyrolysis oil under high hydrogen pressure with a catalyst (Ahamed et al. 2021). The yield and properties of upgraded oil depend on process conditions, such as temperature, pressure, and reactor type (Attia et al. 2020). This method requires high pressure and a significant amount of hydrogen. Hydrogen could be supplied from the steam reforming of pyrolysis oil or gaseous products. Steam reforming is a method that produces synthetic gas (syngas) containing rich hydrogen and carbon monoxide as a target product (Zhang et al. 2019). It is usually performed at high temperatures (700–1000 °C) with nickel as a catalyst (Zhang et al. 2013).

Meanwhile, biological upgrading methods may also be applied besides the thermochemical or catalytic upgrading routes mentioned above. It could transform pyrolysis oil into value-added products under mild conditions through microbial conversions. The feedstocks for these conversions could be whole pyrolysis oil as well as separated or hydrolyzed pyrolysate (Doddapaneni and Kikas 2020).

## Bioconversion of the aqueous phase of pyrolysis oil into renewable chemicals and fuels

The most significant factor hindering the direct utilization of pyrolysis oil is water and a hydrophilic fraction dissolved therein, generated as a pyrolysis product. Pyrolysis oil can be separated into two phases (organic and aqueous) by fractionated condensation or spontaneous separation during storage (Arnold et al. 2017). The organic phase can be directly upgraded to improve the properties of fuels and chemicals through various refining technologies (Chan et al. 2020). In contrast, the aqueous phase is challenging to upgrade to valuable products (Mortensen et al. 2011; Bridgwater 2012; Wang et al. 2013), as it contains various undesirable compounds, which are the leading cause of deterioration of the physical properties of the products and prevent metal catalysts from applying to upgrading processes. To remove impurities and recover valuable aqueous products, water extraction or aqueous two-phase extraction could be applied (Song et al. 2009; Vitasari et al. 2011). On the other hand, the aqueous phase contains microbial substrates like cellobiosan, levoglucosan, acetic acid, and glycolaldehyde, which can be converted into value-added products (lipids, ethanol, organic acids, etc.) via biological conversions (Islam et al. 2015; Xu et al. 2020). These conversions may occur either by wild or genetically engineered microorganisms, which can assimilate pyrolytic products and tolerate toxic compounds. According to the literature reported until recently, yeasts or fungi have been the main microorganisms considering the low pH of the hydrophilic fraction. Also, in most cases, prior to being used as substrates, pretreatment steps have been applied, including hydrolysis, pH adjustment, and fermentation inhibitors removal. We present the results in the literature describing the production of renewable chemicals and fuels from the aqueous phase of pyrolysis oil.

### Microbial lipids

Microbial lipids are of great value due to their uses in the biodiesel and pharmaceutical industries. Various substances in pyrolysis oil can be converted to acetyl-CoA and then synthesized into lipids. Organic acids and anhydrous sugars are the primary raw materials in pyrolysis oil for lipids production. For instance, some studies were performed to produce lipids from pyrolysis-derived acetic acid, which was obtained from fast pyrolysis of mixed softwood, using the microalga *Chlamydomonas reinhardtii* (Liang et al. 2013; Zhao et al. 2015). Luque et al. (2016) produced lipids from the pyrolysates of pinewood using the microalgal strain *Chlorella vulgaris* UTEX 2714. In their study, the substrate for *C. vulgaris* UTEX 2714 was prepared by anhydrosugars hydrolysis and the subsequent detoxification process. Pyrolytic sugars were blended with glucose at different percentages (10, 20, 30, 40, and 50% v/v). The maximum yield of lipid was 0.25 g/g-glucose using a 10% (v/v) glucose blend. A pyrolytic aqueous phase was employed in another study to produce lipids with two yeast strains, *Rhodotorula glutinis* ATCC 204091 and *Cryptococcus curvatus* ATCC 20509. Sulfuric acid was used to hydrolyze the pyrolytic sugars in the aqueous phase into glucose. The hydrolyzed aqueous phase was further neutralized and detoxified with barium hydroxide (Ba(OH)_2_) and activated carbon, respectively. The fermentation of the aqueous phase resulted in lipid yields of 0.089 g/g-glucose and 0.167 g/g-glucose for *R. glutinis* ATCC 204091 and *C. curvatus* ATCC 20509, respectively (Lian et al. 2010). Later, Lian et al. (2012) employed the yeast strain *C. curvatus* ATCC 20509 to produce lipids from the acetate-rich aqueous phase. The aqueous phase was neutralized and detoxified to eliminate its toxic components. The upgraded pyrolytic aqueous phase (20 g/L acetates) was fermented with *C. curvatus* ATCC 20509, yielding 2.2 g/L lipids (36.7% on a dry cell basis). In another study, *R. glutinis* ATCC 204091 was used for lipid production from non-hydrolyzed levoglucosan. The detoxified pyrolytic aqueous phase was fermented by *R. glutinis* ATCC 204091, resulting in biomass and lipid production of 3.3 and 0.78 g/L, respectively (Lian et al. 2013). The bacterial strain *Pseudomonas putida* KT2440 was employed to produce rhamnolipid from organic condensate after solid-phase extraction (OC_SPE_), resulting in a rhamnolipid yield of 0.48 g/g OC_SPE_ (Arnold et al. 2019a).

### Bioethanol

Bioethanol is the most representative biofuel, and it can be a promising feedstock for chemicals such as ethylene. *Saccharomyces cerevisiae*, a typical ethanol producer, cannot assimilate the anhydrous sugar fraction in pyrolysate. Therefore, pretreatment strategies using acid hydrolysis have been proposed to obtain fermentable substrates for the yeast.

Lian et al. (2010) studied ethanol production with the yeast strain *Saccharomyces cerevisiae* ATCC 200062 using a pyrolytic aqueous phase. The aqueous phase was treated sequentially with sulfuric acid, Ba(OH)_2_, and activated carbon for hydrolysis and detoxification. The fermentation process of the resulting aqueous phase achieved an ethanol yield of 0.473 g/g-glucose. Sukhbaatar et al. (2014) reported ethanol production using pinewood pyrolysis oil (aqueous phase) with *Saccharomyces pastorianus* ATCC 2345. The aqueous fraction was hydrolyzed with sulfuric acid, neutralized by sodium hydroxide, and then detoxified with n-butanol. The sugars in the aqueous fraction were concentrated and afterward fermented, leading to a maximum ethanol yield of 98% of the theoretical one.

Islam et al. (2018) employed waste cotton pyrolysate to produce ethanol by *Saccharomyces cerevisiae* 2.399. The hydrolysis and neutralization of pyrolysate were performed using sulfuric acid and Ba(OH)_2_, respectively. After that, ethyl acetate and activated carbon were used to eliminate fermentation inhibitors from the neutralized pyrolysate. In a shake flask experiment, the maximum ethanol concentration of 14.78 g/L was obtained with a hydrolysate glucose concentration of 4%. The ethanol productivity was 0.92 g/L/h. A stirred tank bioreactor with a capacity of 7-L (3 L working volume) was employed in batch fermentation, resulting in 1.32 g/L/h ethanol productivity, which was higher than that of the shake flasks. Maximum ethanol yields were 91% (shake flasks) and 89% (fermenter) of the theoretical one. In a recent study, Basaglia et al. (2021) used the yeast strain *Saccharomyces cerevisiae* L13 to produce ethanol using the H_3_PO_4_-pretreated pyrolytic aqueous phase. *S. cerevisiae* L13 could not grow in a pure or diluted aqueous phase as it could not directly utilize levoglucosan as a carbon source. Phosphoric acid (H_3_PO_4_) was applied to convert levoglucosan into glucose, and *S. cerevisiae* L13 produced around 8.0 g/L of ethanol from the pretreated solution diluted 1:5 (v/v).

However, this strategy requires an additional neutralization step, and fermentation inhibitors such as furfural may be generated during hydrolysis. In this regard, studies on improving microorganisms to utilize levoglucosan directly have also been reported. Layton et al. (2011) expressed the levoglucosan kinase (*lgk*) gene from *Lipomyces starkeyi* YZ-215 in *E. coli* KO11. This strain could consume pure levoglucosan and convert it to ethanol. The yield of ethanol was 0.35 g/g-levoglucosan. In another study, the *lgk* gene from *Aspergillus niger* was expressed in *Saccharomyces cerevisiae* H158 to convert pure levoglucosan into ethanol (Xie et al. 2005). Recently, an inhibitor-tolerant levoglucosan-utilizing strain, *E. coli*-H, was constructed by long-term adaptive evolution (Chang et al. 2021). This strain could produce 8.4 g/L of ethanol from undetoxified bio-oil containing around 2% (w/v) of levoglucosan along with 82% of yield.

### Organic acids

Organic acids are essential chemicals in the pharmaceutical and food industries. In addition, since some organic acids can be used as raw materials for various biodegradable polymers or as solvents in the electronics industry, market demands are growing. They can be obtained from pyrolytic substrates through microbial fermentation. For instance, the fungal strain *Aspergillus terreus* K26 could convert crude levoglucosan, which was crystallized from tar, into itaconic acid (Nakagawa et al. 1984). The fungal strain *Aspergillus niger* CBX-209 was used to produce citric acid from purified levoglucosan, resulting in a citric acid titer of 70 g/L with a yield of 0.875 g/g-levoglucosan (Zhuang et al. 2001). The bacterial strain *E. coli* MG 1655 was employed to produce succinic acid from the pyrolytic aqueous phase of rice husk. The succinic acid titer was 2.42 g/L using a 20% pyrolytic aqueous phase supported with M9 mineral salts and glucose (4 g/L), while it was 0.38 g/L without glucose supplementation (Wang et al. 2013). Yang et al. (2014) developed a two-step bioconversion process using two fungal strains, *Phanerochaete chrysosporium* EBL0511 and *Aspergillus niger* CBX-209 for citric acid production from the aqueous phase in corn stover pyrolysis. First, *P. chrysosporium* EBL0511 was employed to reduce the inhibitor compounds. In the following step, the treated liquid was fermented by *A. niger* CBX-209, and citric acid could be obtained with a yield of 82.1% based on the levoglucosan.

Vardon et al. (2015) used a lignin-derived phenolic liquor to produce muconate using an engineered strain of *Pseudomonas putida* KT2440. This strain could produce 0.70 g/L of muconate in shake flask cultivation. In a recent study, Henson et al. (2021) developed a *Pseudomonas putida* strain to produce muconic acid (MA) and methyl muconic acid (MMA) from catalytic fast pyrolysis wastewater (CFPW). The engineered *P. putida* incorporating about 30 kilobases of genes in the genome could consume 89% (w/w) of carbon in CFPW. These genes encode pathways for utilizing (alkyl)phenols, acetone, and furfural. Through further strain engineering, all aromatic compounds in a simulated CFPW were converted to MA and MMA with ~ 90% (mol/mol) yield. Schmollack et al. (2022) used a genetically engineered *Corynebacterium glutamicum* strain to produce itaconic acid from an acetate-containing aqueous side-stream of fast pyrolysis (AASFP). The titer of itaconic acid was 3.38 g/L. The detoxified pyrolytic aqueous condensate (PAC) was employed for malic acid production by *Aspergillus oryzae* DSM 1863. This strain could produce 9.77 g/L of malic acid (Kubisch and Ochsenreither 2022).

### Other renewable chemicals

**Fig. 2:**
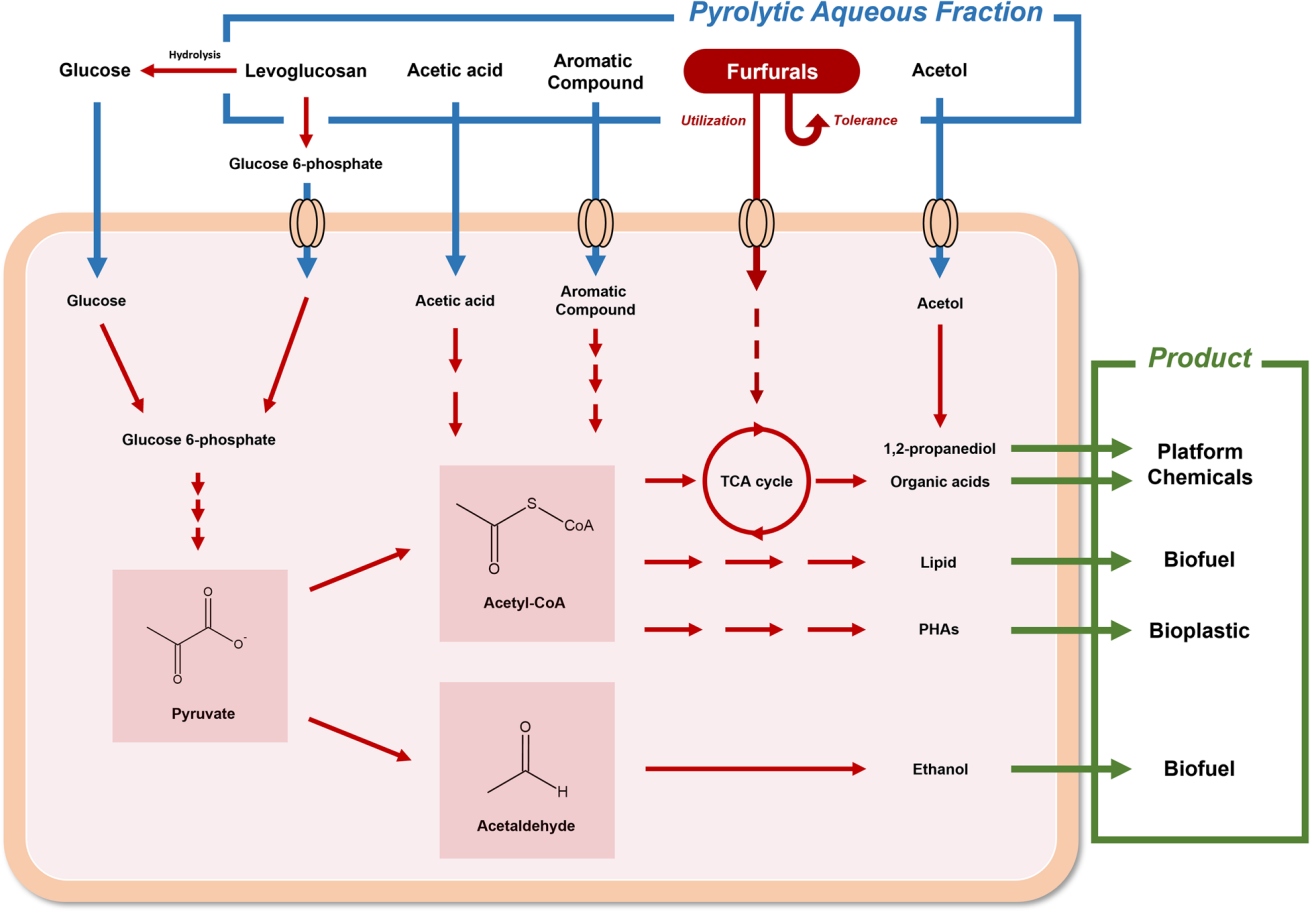
Metabolic pathways of some pyrolytic substrates to produce some value-added products

A few studies were performed to produce other renewable chemicals using pyrolytic substrates present in the aqueous phase. For instance, Linger et al. (2014) reported that the bacterial strain *Pseudomonas putida* KT2440 could produce polyhydroxyalkanoates (PHAs) using phenolic liquor generated from lignin. Lian et al. (2016) expressed a codon-optimized *lgk* gene from *Lipomyces starkeyi* in *E. coli* NST74. The resulting strain could consume pure levoglucosan and convert it to styrene. The titer of styrene was 240 mg/L. Lange et al. (2017) employed a genetically engineered *Corynebacterium glutamicum* strain to produce 1,2-propanediol using the pyrolysis water of wheat straw as substrate. The glycerol dehydrogenase gene *gldA* from *E. coli* was expressed, and the resulting strain could produce 1.39 g/L of 1,2-propanediol with a yield of 0.986 g/g-acetol in fed-batch fermentation with two stages consisting of aerobic and microaerobic conditions. The metabolic pathways of some pyrolytic substrates are summarized in Fig. 2.

Since numerous compounds are in the hydrophilic portion of pyrolysis oil, it may not be practical to produce a single liquid product exclusively. Therefore, methane production by anaerobic digestion of hydrophilic fractions through a microbial consortium could be an option in the short term. The aqueous fraction of the pyrolysis oil from corn stalk pellets was digested to produce methane using various inoculums (Torri and Fabbri 2014). The fed-batch anaerobic digestion of the pyrolytic aqueous phase attained a methane yield of 34% of the theoretical one. The addition of biochar to the anaerobic digestion process increased the methane yield to 60% of the theoretical one, which is attributed to the ability of biochar to remove some of the inhibitor compounds in the pyrolytic aqueous phase. The semi-continuous operation could improve the methane yield to 65% of the theoretical one. Table 4 presents some value-added products generated from pyrolytic substrates through microbial conversions.

**Table 4:** Microbial conversion of pyrolytic substrates into value-added products

Microorganism	Pyrolytic substrate	Product	Titer (g/L)	Yield (g/g)	References
*Chlamydomonas reinhardtii*	Acetic acid	Lipid	~ 0.16	–	(Liang et al. 2013)
*Chlorella vulgaris* UTEX 2714	Anhydrosugars	Lipid	1.59	0.250	(Luque et al. 2016)
*Rhodotorula glutinis* ATCC 204091	Anhydrosugars	Lipid	–	0.089	(Lian et al. 2010)
*Cryptococcus curvatus* ATCC 20509	Anhydrosugars	Lipid	–	0.167	(Lian et al. 2010)
*Cryptococcus curvatus* ATCC 20509	Acetate	Lipid	2.20	~ 0.120	(Lian et al. 2012)
*Rhodotorula glutinis* ATCC 204091	Levoglucosan	Lipid	0.78	0.039	(Lian et al. 2013)
*Pseudomonas putida* KT2440	OC_SPE_	Rhamnolipid	–	0.48	(Arnold et al. 2019a)
*Saccharomyces cerevisiae* ATCC 200062	Anhydrosugars	Ethanol	32.00	0.473	(Lian et al. 2010)
*E. coli* KO11 + *lgk*	Levoglucosan	Ethanol	~ 6.00	0.350	(Layton et al. 2011)
*Saccharomyces pastorianus* ATCC 2345	Levoglucosan	Ethanol	12.12	0.500	(Sukhbaatar et al. 2014)
*Saccharomyces cerevisiae* 2.399	Anhydrosugars	Ethanol	14.78	0.460	(Islam et al. 2018)
*Saccharomyces cerevisiae* L13	Levoglucosan	Ethanol	8.02	0.480	(Basaglia et al. 2021)
*Escherichia coli*-H	Levoglucosan	Ethanol	8.40	0.420	(Chang et al. 2021)
*Aspergillus terreus* K26	Levoglucosan	Itaconic acid	–	0.630	(Nakagawa et al. 1984)
*Corynebacterium glutamicum* ITA13	AASFP	Itaconic acid	3.38	–	(Schmollack et al. 2022)
*Aspergillus niger* CBX-209	Levoglucosan	Citric acid	70.00	0.875	(Zhuang et al. 2001)
*E. coli* MG-PYC	Anhydrosugars	Succinic acid	2.42	–	(Wang et al. 2013)
*Aspergillus niger* CBX-209	Levoglucosan	Citric acid	–	0.821	(Yang et al. 2014)
*Aspergillus oryzae* DSM 1863	PAC	Malic acid	9.77	0.23	(Kubisch and Ochsenreither 2022)
*Pseudomonas putida* KT2440-CJ103	Aromatic compounds	Muconate	0.70	–	(Vardon et al. 2015)
*Pseudomonas putida* KT2440	Aromatic compounds	Polyhydroxyalkanoates	~ 0.25	–	(Linger et al. 2014)
*Corynebacterium glutamicum* PDO2	Acetol	1,2-propanediol	1.39	0.986	(Lange et al. 2017)
*E. coli* NST74	Levoglucosan	Styrene	0.240	0.021	(Lian et al. 2016)

## Challenges and perspectives for the biological upgrading of pyrolysis oil into renewable chemicals and fuels

Although some promising results have been reported, the critical metrics of industrial biotechnology, such as titer and yield, are insufficient for implementation, as shown in Table 4. The main reason for the poor performance is the presence of toxic compounds, such as furfurals, phenolics, and aldehydes, which are well known to inhibit the growth of various microorganisms (Doddapaneni and Kikas 2020). Furanic compounds can deactivate cell reproduction, cause DNA damage, and inhibit the essential enzymes involved in the carbon metabolic pathway (Palmqvist and Hahn-Hagerdal 2000). Phenolic compounds can alter the cell membrane permeability and generate multiple oxygen-free radicals (Monlau et al. 2014; Luo et al. 2021). Aldehyde is also a toxic compound present in the pyrolytic aqueous phase. It can inhibit microbial growth by hindering the formation of cellular membranes, proteins, and DNA (Jayakody et al. 2017). Detoxification and neutralization have been used to solve this problem. However, these methods have the disadvantage of requiring additional steps with large amounts of chemicals. A fundamental solution is to develop microorganisms resistant to toxicity. Various strategies exist for constructing microbial catalysts against toxic substances: adaptive evolution or pathway design and manipulation to insert/remove relevant genes related or labor division by microbial consortium.

The first strategy is to isolate and select some microorganisms that can grow on the pyrolytic aqueous phase and tolerate toxic components. Lee et al. (2016) isolated some bacteria that can utilize furfural from wastewater treatment plants. In another study, Lee et al. (2010) found that *Corynebacterium glutamicum* ATCC 13032 could decompose phenol of 0.8 g/L and the presence of phenol in the culture medium enhanced the production of glutamate and proline. Hasan and Jabeen (2015) isolated two bacterial strains, *Bacillus subtilis* and *Pseudomonas* sp., which could tolerate phenol. Singh et al. (2017) isolated *Bordetella* sp. BTIITR from soil that could remove 100% of furfural and 94% of 5-hydroxymethylfurfural from sugarcane bagasse hydrolysate after an incubation period of 16 h. Zheng et al. (2015) reported that *Bacillus subtilis* DS3 could metabolize furfural as a sole carbon source. It can degrade 31.2% furfural and tolerate 6 g/L furfural. Bhatia et al. (2019) demonstrated that the bacterial strain *Ralstonia eutropha* 5119 can co-metabolize various inhibitors, including furfural and 5-hydroxymethylfurfural, along with glucose for biomass and polyhydroxyalkanoate production.

The genetic engineering of microorganisms could be another strategy to enhance their tolerance to those toxic substances and enable them to transform into value-added products. Furfural utilization is the main target, and some successful results have been reported. In this respect, the furfural resistance of *E. coli* LY180 was improved by deleting the NADPH-dependent oxidoreductase genes (*yqhD* and *dkgA*) (Miller et al. 2009). Also, the overexpression of the NADH-dependent propanediol oxidoreductase gene (*fucO*) in *E. coli* LY180 increased its resistance to furfural (Wang et al. 2011). In a subsequent study, Zheng et al. (2012) expressed the thymidylate synthase (*thyA*) gene from *Bacillus subtilis* in *E. coli* to enhance its tolerance to furfural. Jiménez-Bonilla et al. (2020) overexpressed the solvent-resistant pump (*srpB*) gene from *Pseudomonas putida* in *Clostridium saccharoperbutylacetonicum* N1-4 to improve its tolerance to furfural. The engineered strain could grow in media containing up to 3.5 g/L furfural. Jayakody et al. (2018) found that integrating an extra copy of the NADPH-dependent methylglyoxal reductase (*GRE2*) gene in an engineered *Saccharomyces cerevisiae* strain could increase its tolerance to glycolaldehyde.

Mixed fermentation could be an option for mitigating the toxicity of the pyrolytic aqueous phase and facilitating the co-production process (Kim et al. 2020). A combination of microorganisms has been applied to consume various pyrolytic substrates concurrently or sequentially (Yang et al. 2014; Doddapaneni and Kikas 2020). In a recent study, *Fusarium striatum* UdL-TA-3.335 and *Saccharomyces cerevisiae* CEN.PK XXX were co-cultured on wheat straw hydrolysate containing high concentrations of furfural and 5-hydroxymethylfurfural. The fungus *F. striatum* could reduce the hydrolysate toxicity, enhancing the fermentation performance of *S. cerevisiae.* An ethanol yield of 0.4 g/g was attained in a 1.5-L bioreactor containing furfural (2.5 g/L) and 5-hydroxymethylfurfural (3.5 g/L) (Acosta et al. 2021).

Another cause for the poor bioconversion performance, including low product titer and substrate conversion, is the low spectrum for substrate utilization. For the full-fledged biological application of pyrolysis oil, it is necessary to utilize a wide range of substrates in the aqueous phase. In the previous examples, most substrates were limited to levoglucosan or some organic acids. Recently, there have been several reports for the assimilation of unconventional carbon sources, such as aldehydes and organic acids, although not for upgrading the pyrolysis oil.

Lu et al. (2019) designed and constructed a synthetic pathway by engineering glycolaldehyde synthase and acetyl-phosphate synthase. Through this strategy, the engineered strain containing the novel pathway could grow on 0.4 g/L of glycolaldehyde, which is one of the most abundant substances in the hydrophilic fraction of pyrolysis oil. Also, metabolic engineering approaches and cultivation strategies have been reported to utilize organic acids, such as glycolate, acetate, butyrate, and levulinate as the sole carbon source (Clark and Cronan 1996; Huang et al. 2016; Habe et al. 2020; Park et al. 2020). Organic acids can be incorporated into central metabolism via acetyl-CoA and then converted into various products. Bang and Lee (2018) reported the assimilation of formate and CO_2_ as sole carbon sources for engineered *E. coli* by reconstructing the tetrahydrofolate cycle and creating the reverse glycine cleavage route along with heterologous formate dehydrogenase introduction.

**Fig. 3:**
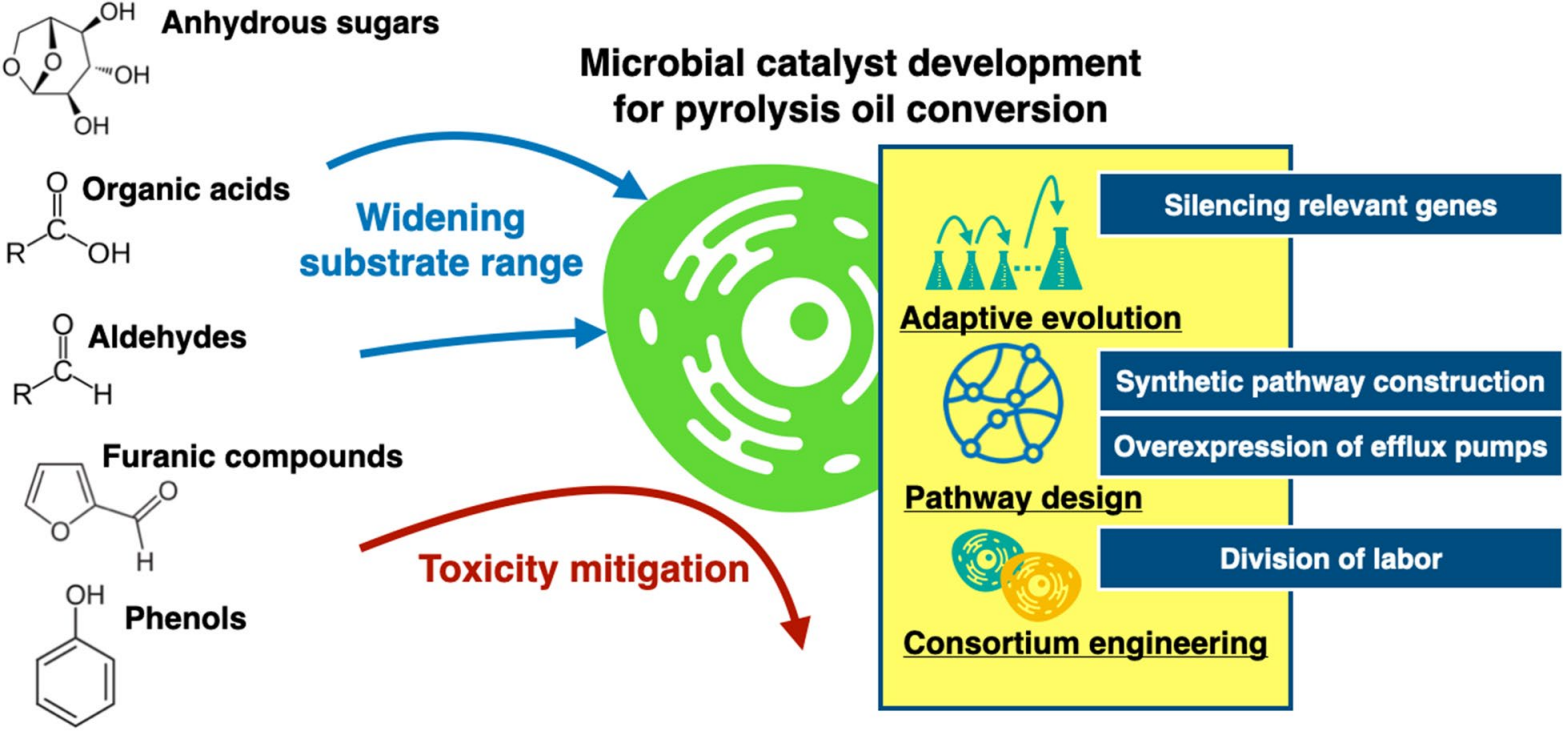
Development strategies of microbial catalysts to utilize the hydrophilic fraction of pyrolysis oil

On the other hand, the strategy to co-metabolize various carbon sources concurrently would be developed. In this respect, it is necessary to address the catabolite repression issue in the presence of multiple substrates using genetic tools to control the regulation of carbon source assimilation. The engineered microbial consortium can also give the solution for this topic. Valorizing the pyrolysis oil can be achieved by mixed culture in a controlled manner. The strategies for improving the performance of the bioupgrading method are summarized in Fig. 3.

The points mentioned above mainly concern the efficient use of produced pyrolysis oil. However, to mass-produce components suitable for microbial metabolism, selecting suitable biomass for upgrading the aqueous fractions in the upstream pyrolysis process or minimizing the degradation of microbial substrates during thermal conversion is also worth considering.

## Conclusions

Pyrolysis oil is a promising feedstock to produce renewable chemicals and fuels through microbial upgrading. It can be separated into organic and aqueous phases. The presence of diverse toxic compounds and the complex composition of the aqueous phase are the main challenges to its conversion into renewable chemicals and fuels. Microbial strains that can efficiently assimilate pyrolytic substrates and tolerate toxic compounds are needed to face these challenges. Also, further studies are still required to optimize process conditions and increase process performance in terms of product titer and yield.

## Data Availability

Not applicable.

## Abbreviations

LEDS: Low emission development strategies
CO_2_: Carbon dioxide
GC: Gas chromatography
GC × GC: Two-dimensional GC
Py-GC/MS: Pyrolysis GC–mass spectrometry
LC: Liquid chromatography
HRMS: High-resolution MS
NMR: Nuclear magnetic resonance
FTIR: Fourier transform infrared spectroscopy
OC_SPE_: Organic condensate after solid-phase extraction
AASFP: Acetate-containing aqueous side-stream of fast pyrolysis
PAC: Pyrolytic aqueous condensate
Ba(OH)_2_: Barium hydroxide
H_3_PO_4_: Phosphoric acid
Lgk: Levoglucosan kinase
MA: Muconic acid
MMA: Methyl muconic acid
CFPW: Catalytic fast pyrolysis wastewater
PHAs: Polyhydroxyalkanoates
GldA: Glycerol dehydrogenase
ThyA: Thymidylate synthase
SrpB: Solvent-resistant pump
GRE2: Methylglyoxal reductase
